# MCMTC: A Pragmatic Framework for Selecting an Experimental Design to Inform the Development of Digital Interventions

**DOI:** 10.3389/fdgth.2022.798025

**Published:** 2022-03-09

**Authors:** Inbal Nahum-Shani, John J. Dziak, David W. Wetter

**Affiliations:** ^1^Insitute for Social Research, University of Michigan, Ann Arbor, MI, United States; ^2^Edna Bennett Pierce Prevention Research Center, The Pennsylvania State University, State College, PA, United States; ^3^Huntsman Cancer Institute, University of Utah, Salt Lake City, UT, United States

**Keywords:** Sequential Multiple Assignment Randomized Trial (SMART), Micro-Randomized Trial (MRT), factorial designs, adaptive interventions, just in time adaptive interventions, digital interventions

## Abstract

Advances in digital technologies have created unprecedented opportunities to deliver effective and scalable behavior change interventions. Many digital interventions include multiple components, namely several aspects of the intervention that can be differentiated for systematic investigation. Various types of experimental approaches have been developed in recent years to enable researchers to obtain the empirical evidence necessary for the development of effective multiple-component interventions. These include factorial designs, Sequential Multiple Assignment Randomized Trials (SMARTs), and Micro-Randomized Trials (MRTs). An important challenge facing researchers concerns selecting the right type of design to match their scientific questions. Here, we propose MCMTC – a pragmatic framework that can be used to guide investigators interested in developing digital interventions in deciding which experimental approach to select. This framework includes five questions that investigators are encouraged to answer in the process of selecting the most suitable design: (1) Multiple-component intervention: Is the goal to develop an intervention that includes multiple components; (2) Component selection: Are there open scientific questions about the selection of specific components for inclusion in the intervention; (3) More than a single component: Are there open scientific questions about the inclusion of more than a single component in the intervention; (4) Timing: Are there open scientific questions about the timing of component delivery, that is when to deliver specific components; and (5) Change: Are the components in question designed to address conditions that change relatively slowly (e.g., over months or weeks) or rapidly (e.g., every day, hours, minutes). Throughout we use examples of tobacco cessation digital interventions to illustrate the process of selecting a design by answering these questions. For simplicity we focus exclusively on four experimental approaches—standard two- or multi-arm randomized trials, classic factorial designs, SMARTs, and MRTs—acknowledging that the array of possible experimental approaches for developing digital interventions is not limited to these designs.

## Introduction

The widespread use, acceptability and convenience of digital technologies (e.g., mobile and wearable devices) have the potential to reduce structural barriers to treatment, making possible the delivery of behavioral interventions anytime and anywhere (1–3). Many digital interventions include multiple components. A component is any aspect of an intervention that can be separated out for systematic investigation (4). Examples of components in digital interventions include different content modules (5), levels of content tailoring [e.g., the degree to which information about the individual or context is used to produce the message (6)], intensity of human support (7), as well as other features designed to promote engagement such as gamification [i.e., the use of game-design elements in a non-game context (8)] and reminders (9). In their review of text messaging-based smoking cessation interventions, Kong et al. (10) conclude that to develop effective digital interventions to support tobacco cessation “we need to broaden our understanding of the specific components of the interventions, for whom and how they can be used, and identify areas to improve already existing interventions”.

Various types of experimental approaches have been developed in recent years to enable researchers to obtain the empirical evidence necessary for the development and/or evaluation of effective multiple-component interventions. These include standard two- or multi-arm randomized trials [e.g., randomized controlled trials (RCTs)], factorial designs (11, 12), Sequential Multiple Assignment Randomized Trials [SMARTs (13, 14)], and Micro-Randomized Trials [MRTs (15, 16)]. An important challenge facing researchers interested in developing digital interventions concerns the selection of an appropriate experimental design to achieve their scientific goals. Here, we propose MCMTC, a pragmatic framework that can be used to guide investigators interested in developing digital interventions in deciding which experimental approach to select based on their scientific questions. MCMTC can be used not only as a guide for investigators considering potential studies, but also as a framework for teaching students the practical differences between various experimental design options.

## The MCMTC Pragmatic Framework

**Figure 1:**
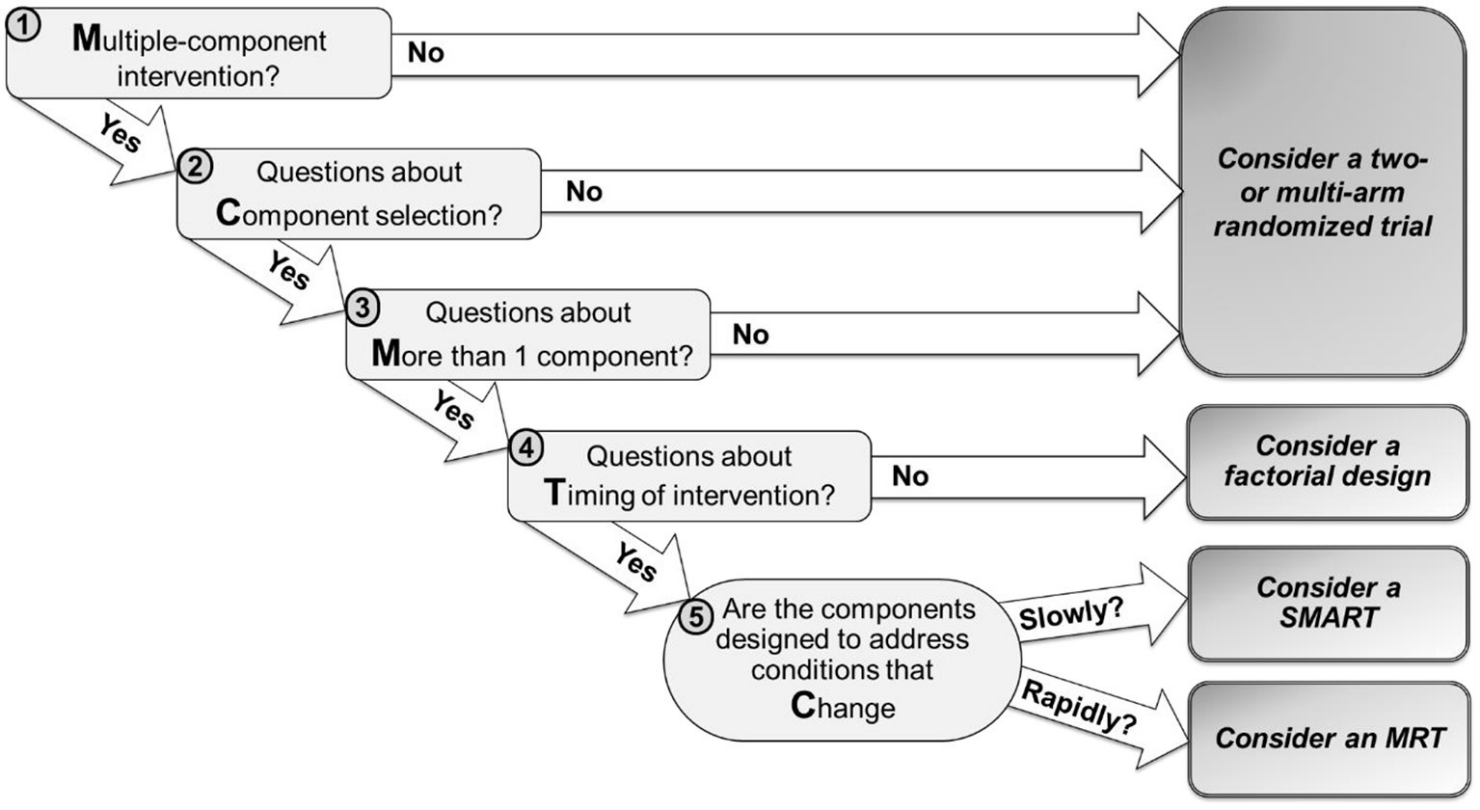
MCMTC: a pragmatic framework for selecting an experimental design to inform the development of digital interventions.

The MCMTC framework includes five questions that investigators can follow to guide their decision (Figure 1). In the next sections, we discuss each question using examples of tobacco cessation digital interventions to illustrate how answering these questions can guide the choice of a specific design. While a wide variety of study designs can inform the development of digital interventions, this manuscript focuses on four experimental approaches: standard two- or multi-arm randomized trials, classic factorial designs, SMARTs, and MRTs.

### Question 1: Multiple-Component Intervention?

The first question is whether investigators wish to develop an intervention that includes multiple components.

#### No: The Goal Is Not to Develop a Multiple-Component Intervention

Examples where the answer to Question 1 is “No” include interventions either with a single component or else with multiple elements that should not be differentiated from each other for investigation. For example, suppose investigators develop a new type of nicotine replacement therapy (NRT) to support smoking cessation. A natural next step is to evaluate the effectiveness of this new NRT relative to a suitable alternative (e.g., the standard of care or another existing product). In this case, a randomized controlled trial (RCT) comparing the single component intervention to control would be a suitable experimental design. Next, suppose investigators develop a new digital intervention to support tobacco cessation which includes phone coaching combined with text messaging to help coordinate and schedule the calls with participants. This intervention includes two elements, phone coaching and text messaging, but these elements cannot be practically separated out for investigation because they are implemented in an interdependent way (i.e., text messaging to schedule calls could not be a standalone intervention given it supports phone coaching). Alternatively, suppose that the text messaging element focuses instead on encouraging participants to use brief self-regulatory strategies (e.g., deep breathing, calling a friend for support or taking a walk). In this case, text messaging and phone coaching can be separated out for investigation as they are not implemented in an interdependent way. However, suppose there is empirical evidence indicating that these two elements work together synergistically and hence should be combined, rather than implemented separately, to effectively promote tobacco cessation. In both cases, a natural next step investigators may take is to evaluate the combined effectiveness of the two elements, relative to a suitable alternative (e.g., standard of care). Hence, an RCT comparing the integration of coaching with text messaging to control may be a suitable experimental design.

Notice that in the examples above the goal is not to empirically develop (i.e., systematically assemble the elements of) an intervention, but rather to evaluate the effectiveness of an intervention that either includes a single element or multiple elements. In these cases, investigators may consider a standard RCT comparing the effectiveness of the intervention as a whole to a suitable alternative. However, when the goal is to develop an intervention by investigating the effect of multiple components in order to find a favorable combination, a more complex design may be needed.

#### Yes: The Goal Is to Develop a Multiple-Component Intervention

Examples where the answer to Question 1 is “Yes” include cases where specific aspects of the intervention can be separated out for investigation. For example (*Example A*), suppose investigators wish to develop a digital tobacco cessation intervention and they consider three elements: (1) a mobile app containing a collection of on-demand mindfulness-based meditation activities (Mobile); (2) daily prompts (*via* text messaging) encouraging participants to use brief self-regulatory strategies, such as deep breathing, calling a friend for support or taking a walk (Prompts); and (3) weekly online sessions containing brief quitting advice (Sessions). Suppose these elements can be feasibly separated out for investigation. In this case, the answer to Question 1 would be “Yes”, leading investigators to Question 2.

### Question 2: Component Selection?

The second question is whether there are open scientific questions about the selection of specific components for inclusion in the intervention.

#### No: There Are No Open Scientific Questions About the Selection of Components

Examples where the answer to Question 2 may be “No” include cases where investigators already have sufficient evidence (empirical or practical) to decide which components to include in the intervention. Suppose the investigators from the previous example have sufficient empirical evidence to conclude that all three components—Mobile, Prompts and Sessions—should be included in the intervention. If there are no questions about the efficacy of these components or their interactive effects, a natural next step would be to put together an intervention package that contains all three components and conduct an RCT to evaluate the effectiveness of the combined package, relative to a suitable control. In sum, MCMTC recommends that in the absence of scientific questions about the selection of intervention components, investigators may focus on scientific questions that concern the effectiveness of the multiple-component intervention package compared to a suitable alternative and hence consider a standard RCT.

#### Yes: There Are Open Scientific Questions About the Selection of Components

Examples where the answer to Question 2 is “Yes” include cases where there is insufficient evidence to decide which component to include in the intervention package. For example (*Example B*), suppose investigators have sufficient evidence to conclude that the component Mobile should be included in the intervention package, but there is insufficient evidence to decide whether Prompts and Sessions should be included. Here, the investigators pose the following scientific questions (1) should a digital tobacco cessation intervention that includes Mobile also include Prompts? and (2) should a digital tobacco cessation intervention that includes Mobile also include Sessions? This means that there are open scientific questions about the selection of specific components for inclusion in the intervention. Hence, the answer to Question 2 would be “Yes”, leading investigators to Question 3.

### Question 3: More Than One Component?

The third question is whether there are open scientific questions about the inclusion of more than a single component in the intervention.

#### No: There Are No Open Scientific Questions About the Inclusion of More Than One Component

Examples where the answer to Question 3 may be “No” include cases where investigators consider multiple components for inclusion in the intervention package, but their scientific question is about only one of the components. For example, suppose investigators have sufficient evidence to conclude that the components Mobile and Prompts should be included in the intervention package, but there is insufficient evidence to decide whether Sessions should be included. Here, the investigators pose the following scientific question should a digital tobacco cessation intervention that includes Mobile and Prompts, also include Sessions? This question can be addressed *via* a two-arm randomized trial comparing the digital intervention with Sessions (i.e., an intervention package including all three components: Mobile, Prompts and Sessions) to a digital intervention without Sessions (i.e., an intervention package with only two components: Mobile and Prompts).

Examples where the answer to Question 3 is “No” may also include cases where investigators consider multiple components, but their scientific question is about choosing a single one out of multiple components. For example, suppose investigators pose the following question is it most beneficial to offer Mobile, Prompt or Sessions? This question can be addressed by conducting a three-arm trial where participants are randomized to one of these three components. There might optionally also be a control arm receiving none of the components, so that the effectiveness (and not only the relative effectiveness) of each component can be investigated. However, in either case, combinations of components are not considered in this design.

In sum, MCMTC recommends that when there are open scientific questions only about the selection of a single component for inclusion in a multiple-component intervention, or the selection of a single component out of multiple components (but not about combinations of these components), then investigators may consider a standard two- or multi-arm randomized trial.

#### Yes: There Are Open Scientific Questions About the Inclusion of More Than One Component

Examples where the answer to Question 3 is “Yes” include cases where there is insufficient evidence to make decisions about the inclusion of two or more intervention components. Consider *Example B* discussed above, where investigators pose scientific questions about the inclusion of two components, Prompts and Sessions. Here, there are open scientific questions about the inclusion of more than a single component. Hence, the answer to Question 3 would be “Yes”, leading investigators to Question 4.

### Question 4: Timing of Intervention?

The fourth question is whether investigators wish to address scientific questions about the timing of delivering intervention components. These questions concern when it is best to deliver an intervention component or which component is most beneficial at different points in time.

#### No: There Are No Open Scientific Questions About Intervention Timing

Examples where the answer to this question may be “No” include cases where investigators have scientific questions only about which intervention component to include throughout the study, or else to introduce at a single specific point in time. Consider *Example B* discussed above, where investigators pose two questions about the selection of two components at the beginning of the intervention. As an example, consider the first question: should a digital tobacco cessation intervention that includes Mobile also include Prompts? This question is about which component to introduce at a single time point—at study outset. This question concerns neither when to deliver these components, nor which component would be most beneficial at different time points. Since the questions posed in *Example B* do not concern intervention timing, the answer to Question 4 would be “No”, leading investigators to consider a classic factorial design.

##### Classic Factorial Designs

A factorial design is a randomized trial that includes two or more factors [i.e., independent variables manipulated in a systematic manner (4)]. For simplicity, suppose that each factor includes two levels: On (when the corresponding component is present) and Off (when the corresponding component is not present). In a factorial design, the levels of each factor are crossed with the levels of the other factors to form a design with multiple experimental conditions. Consider *Example B* discussed above, where investigators have scientific questions about the inclusion of two components, Prompts and Sessions. To answer these questions, the investigators may consider a factorial experiment with two factors, one factor for each component. Using italicized abbreviations to represent experimental factors, *Prompts* refers to the factor corresponding to daily prompts *via* text messaging, and *Sessions* refers to the factor corresponding to weekly online sessions. Each factor will have two levels On and Off.

In the factorial design presented in Table 1, the two levels of *Prompts* are crossed with the two levels of *Sessions* to form a design with 2 ×2 = 4 experimental conditions. Here, suppose that 400 individuals enter the study (throughout we assume no attrition for simplicity) and are randomized with equal probability (0.25) to each of the four experimental conditions. Suppose the primary outcome of interest was measured at the month 6 follow up. Data from this experimental design can be used to answer the two motivating questions about the inclusion of Prompts and Sessions by testing the main effect of each corresponding factor. When a factor has two levels, the main effect of this factor can be defined as the difference between the mean outcome at one level of this factor and the mean outcome at the other level of this factor, averaging over the levels of the other factors. Using data from the factorial experiment in Table 1, the main effect of *Prompts* can be estimated by comparing the mean outcome across all the experimental conditions in which *Prompts* was set to On (conditions 1 and 2; *n* = 200; Table 1) to the mean outcome across all the conditions in which *Prompts* was set to Off (conditions 3 and 4; *n* = 200; Table 1). Similarly, the main effect of *Sessions* can be estimated by comparing the mean outcome across the experimental conditions in which *Sessions* was set to On (conditions 1 and 3; *n* = 200; Table 1) to the mean outcome across the conditions in which *Sessions* was set to Off (conditions 2 and 4; *n* = 200; Table 1).

**Table 1:** 2 ×2 factorial *N* = 400.

**Experimental condition**	**Factor**	
	**Prompts**	**Sessions**
1 (*n* = 100)	On	On
2 (*n* = 100)	On	Off
3 (*n* = 100)	Off	On
4 (*n* = 100)	Off	Off

Notice that both main effects are estimated by using outcome information from the entire sample (*N* = 400). This is because factorial designs enable investigators to use outcome data from each study participant to test more than one main effect, thereby answering multiple scientific questions about the selection of intervention components. Collins et al. (11) described this property as the “recycling” of study participants and discussed the efficiency of this approach in estimating both main effects and interactions [also see (4, 17)].

Various types of factorial designs and analytic methods have been developed to accommodate scenarios where the implementation of a large number of experimental conditions is not practically feasible (4, 18) and where experimental subjects are clustered prior to the study [e.g., students in schools (19)], or become clustered during the study [e.g., study participants assigned to support groups; (20, 21)]. These methods enable investigators to leverage classic factorial designs to inform the development of a wide variety of behavioral interventions.

#### Yes: There Are Open Scientific Questions About Intervention Timing

**Figure 2:**
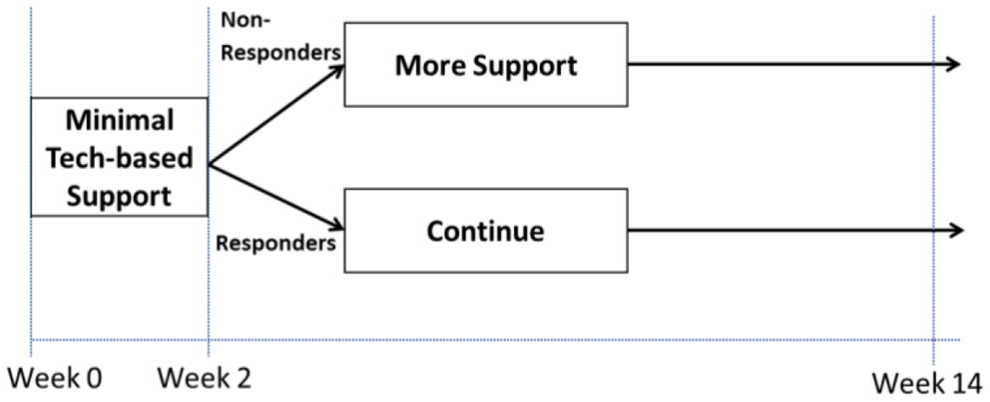
Hypothetical adaptive intervention (Example C).

Examples where the answer to Question 4 may be “Yes” include cases where investigators have scientific questions about when it would be best to deliver a specific intervention component or which component to deliver at different points in time. As an example (*Example C*; see Figure 2), suppose an investigator wishes to develop a tobacco cessation intervention that begins with minimal technology-based support and then at week 2 provides more support to individuals who show early signs of non-response (i.e., those who self-report tobacco use within the past 7 days), whereas early responders (i.e., those who self-report no tobacco use within the past 7 days) continue with minimal technology-based support. Suppose that two scientific questions motivate the investigator: (1) should the initial intervention include a mobile app with a collection of on-demand mindfulness-based meditation activities (Mobile), or the mobile app combined with daily prompts recommending brief self-regulatory strategies (Mobile + Prompts)? (2) Should early non-responders be offered two online sessions containing brief quitting advice (Sessions) or more frequent weekly sessions (Sessions+)? These questions concern intervention timing as they focus on which component should be offered at different points in time—at study outset (week 0) and at week 2. Hence, the answer to Question 4 would be “Yes”, leading investigators to Question 5.

### Question 5: Change Slowly or Rapidly?

The last question is whether the components in question are intended to address conditions that change relatively slowly (e.g., over months or weeks) or rapidly (e.g., every day, hours, minutes)?

#### Slowly: Components Address Conditions That Change Slowly

Examples where the answer to Question 5 may be “slowly” include cases where investigators are interested in developing an adaptive intervention (ADI) and they need to answer questions about the selection and adaptation of components in this intervention. ADIs are interventions that use dynamic (time-varying) information about the person's progress in the course of an intervention (e.g., early response status, adherence) to decide which intervention component to deliver at different decision points (22, 23). Each decision point represents a point in time in which a decision should be made in practice about whether and how to modify the intervention based on what is known about the individual's status and progress. Here, the term “adaptation” refers to the use of dynamic information about the person to decide whether and how to intervene. In the hypothetical example in Figure 2, information about response status (operationalized in terms of whether or not the individual self-reported tobacco use within the past 7 days) is used to decide who should be offered more support and who should continue with the initial intervention. This adaptation process is initiated at week 2 because it is intended to address early signs of non-response, which are expected to unfold (in this example) over 2 weeks. The underlying assumption is that offering more support to those who show early signs of non-response at week 2 would prevent their likely failure to achieve long-term abstinence, whereas those who show early signs of response at week 2 are likely to succeed ultimately and hence would benefit from continuing with the same initial intervention.

More generally, the term ADI usually refers to interventions whose adaptation addresses conditions that change relatively slowly (e.g., over weeks or months). The adaptation is designed to increase the ultimate effectiveness of the intervention (by delivering the type/amount of intervention needed, when it is needed), while reducing cost and burden (by avoiding unnecessary treatment) (23–25). While technology offers tremendous potential for delivering ADIs, empirical data is often needed to inform the selection and adaptation of intervention components, and these knowledge gaps can motivate scientific questions for randomized studies [e.g., (26–30)]. Consider *Example C* discussed above where there are two open scientific questions concerning the development of an ADI: (1) at week 0, should the intervention include only Mobile, or Mobile + Prompts? (2) at week 2, should early non-responders be offered Sessions, or Sessions+? These questions concern which component(s) to deliver at different decision points, in an intervention that intends to address conditions that change relatively slowly. Hence, as suggested in Figure 1, investigators may consider a SMART design.

##### Sequential Multiple Assignment Randomized Trial Designs

**Figure 3:**
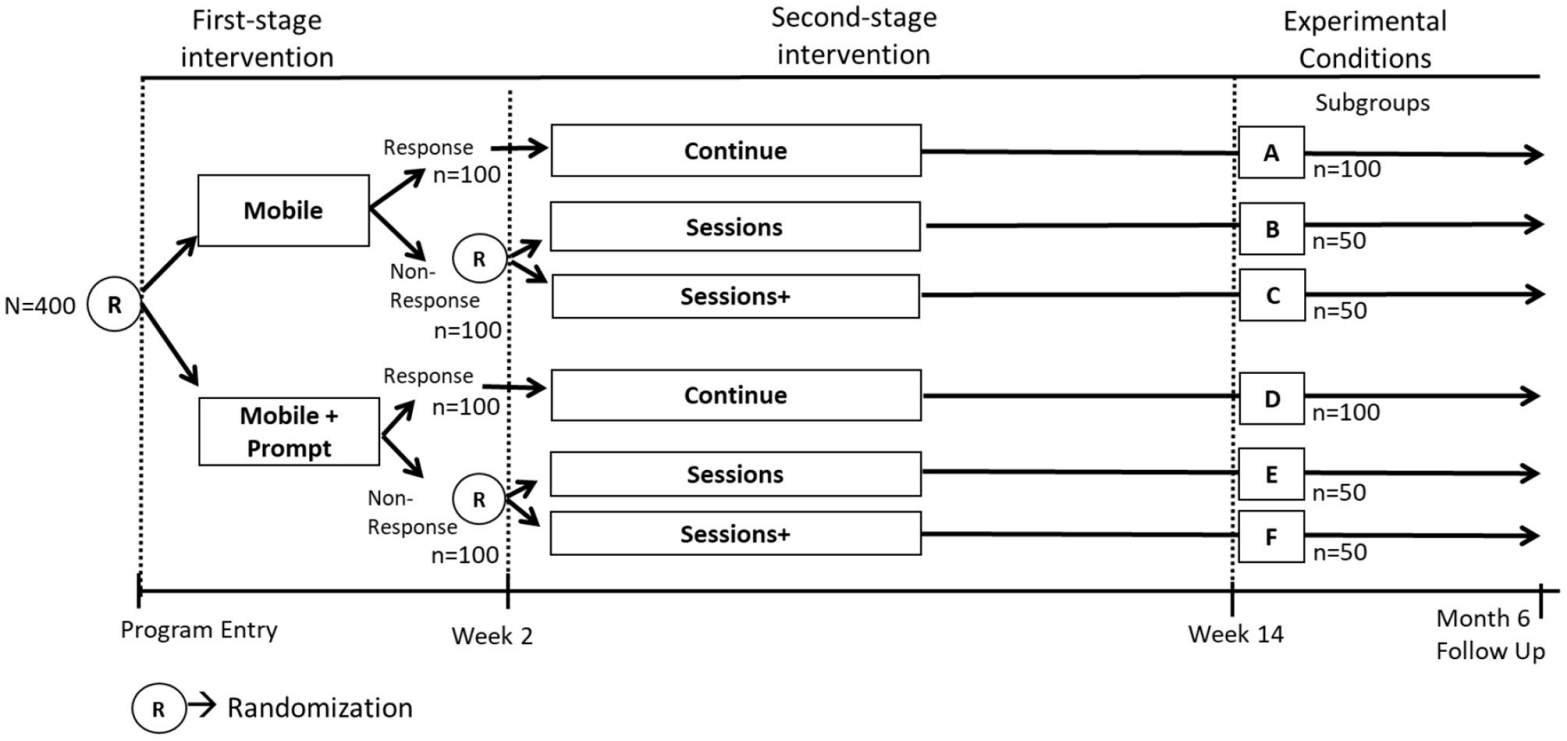
SMART study to answer example C scientific questions.

A SMART is a randomized trial that includes sequential randomizations (13, 14); that is, some or all of the study participants are randomized more than once in the course of the study (31). Each randomization in a SMART is designed to answer scientific questions about the selection and adaptation of components at a specific decision point with the goal of empirically informing the development of an ADI. Consider again the two scientific questions outlined in *Example C*. The first question about which component to offer at week 0 can be answered by randomizing individuals at week 0 to either Mobile or Mobile+Prompt. The second question about which component to offer early non-responders at week 2 can be answered by re-randomizing early non-responders at week 2 to either Sessions or Sessions+. Responders do not get re-randomized and instead continue with the initial intervention. These sequential randomizations result in six experimental conditions, labeled A through F (Figure 3).

Suppose that 400 individuals enter the study and that they are randomized with equal probability (0.5) at week 0. Also, for simplicity suppose that 50% of the participants become classified as non-responders. Suppose that non-responders are re-randomized at week 2 with equal probability (0.5) to the two subsequent components. As before, suppose that the primary outcome of interest is measured at the month 6 follow up. Figure 3 shows the number of participants in each experimental condition A-F based on these assumptions. Similar to the analysis described for *Example B*, the analyses for addressing the scientific questions in *Example C* leverage outcome information across multiple experimental conditions. Specifically, the first question can be answered by comparing the mean outcome across all the experimental conditions in which participants were offered Mobile initially (conditions A, B and C; *n* = 200; Figure 3) to the mean outcome across all the conditions in which participants were offered Mobile+Prompts initially (conditions D, E and F; *n* = 200; Figure 3). Notice that as before, this would involve using outcome data from the entire sample to estimate the effect, which can be viewed as the main effect of the initial component averaging over the subsequent components for responders and non-responders. The second scientific question can be answered by comparing the mean outcome across the two experimental conditions in which early non-responders were offered Sessions subsequently (conditions B and E; *n* = 100; Figure 3) to the mean outcome across the two conditions in which early non-responders were offered Sessions+ subsequently (conditions C and F; *n* = 100; Figure 3). Notice that this comparison would involve using outcome data from the entire sample of early non-responders to estimate the effect, so it can be viewed as the main effect of the subsequent components among early non-responders, averaging over the initial components.

Notice that similar to the classic factorial design discussed earlier, the SMART enables investigators to use outcome data from each study participant to test more than one main effect, thereby answering multiple scientific questions about the selection of components. Hence, SMART designs share a somewhat similar “recycling” property as classic factorial designs.

In recent years, various types of SMART designs and analytic methods have been developed to enable investigators to address specific scientific questions about the selection and adaptation of components. Examples include questions about the differences between ADIs that are embedded in a SMART, and the selection of the best embedded ADI [e.g., (22, 32, 33)], how well components that are offered at different time points work together [e.g., (25, 34)], and what type of information should be used to decide which component to offer [e.g., (35–38)]. These methods enable investigators to leverage the SMART to inform the development of a wide variety of ADIs.

#### Rapidly: Components Address Conditions That Change Rapidly

Examples where the answer to Question 5 may be “rapidly” include cases where investigators are interested in developing a just in time adaptive intervention (JITAI) and they need to answer questions about the selection and adaptation of components in this intervention. A JITAI employs adaptation to address conditions that change relatively rapidly (e.g., every few days, hours, or minutes) and in the person's natural environment (39, 40). Similar to ADIs, the adaptation in JITAIs is designed to enhance the effectiveness of the intervention and to reduce burden by delivering the type/amount of intervention needed, only to those who need it, and only when they need it. However, because JITAIs are intended to address conditions that change rapidly and in daily life, where multiple demands compete for the person's attention and effort, the adaptation is also explicitly intended to minimize disruptions to the daily lives of individuals (40, 41).

**Figure 4:**
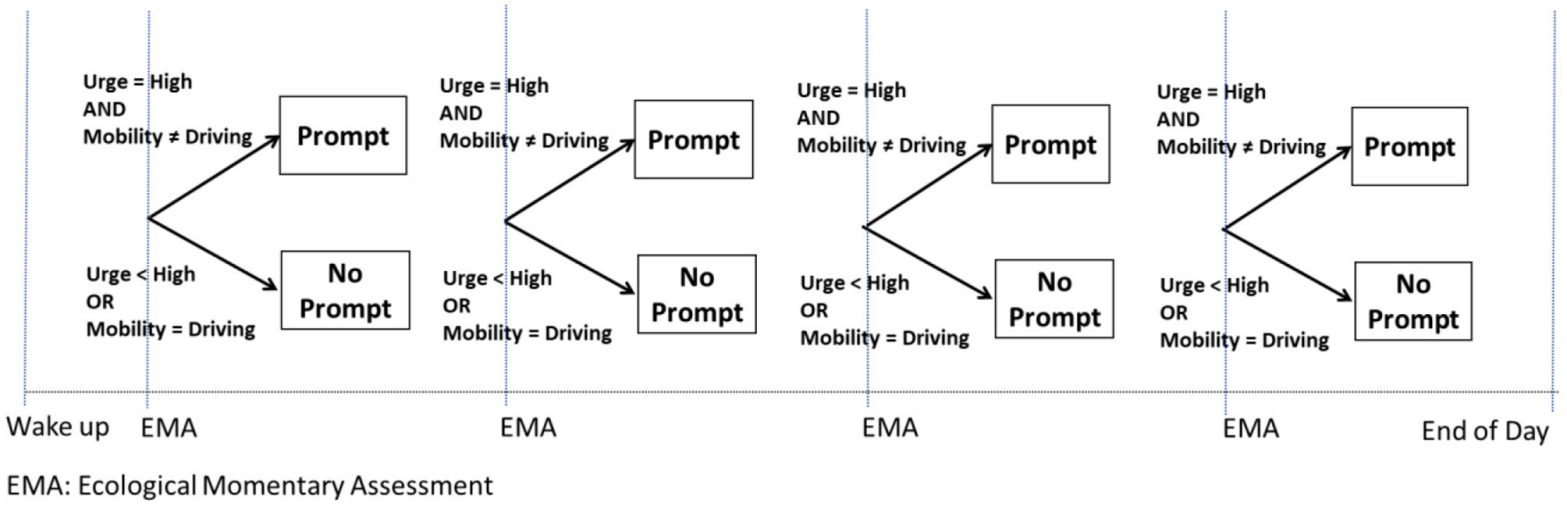
Hypothetical just in time adaptive intervention (Example D).

As an example (*Example D*), consider a JITAI for preventing a smoking lapse by delivering a mobile-based prompt [e.g., *via* a push notification from a mobile app; see (42)] that recommends a self-regulatory strategy when individuals self-report an urge to smoke and are not driving a car. Participants' urge to smoke is monitored *via* Ecological Momentary Assessments (EMAs) (43) four times per day (triggered at randomly selected times and spread throughout the person's waking hours, with ~3 h between each), and assisted GPS technology is used to track and calculate their minute-by-minute mobility pattern (44–46). If the person self-reports high urge to smoke *via* the EMA and they are not driving a car, a prompt recommending a brief self-regulatory strategy is delivered on their mobile device. Otherwise, a prompt is not delivered (Figure 4). This intervention is adaptive because it uses dynamic information about the person's internal state (i.e., urge to smoke) and context (i.e., mobility pattern) to decide whether and how to intervene. This intervention is a JITAI because the adaptation is intended to address conditions that change rapidly (urge to smoke may emerge multiple times per day, representing risk for a smoking lapse) based on real-world context (urge to smoke may emerge in the person's natural environment, outside of standard treatment settings). The adaptation in this hypothetical JITAI is intended not only to prevent a smoking lapse (by addressing high urge to smoke) while avoiding unnecessary intervention (not delivering an intervention when urge to smoke is less than high), but also to minimize disruptions to the daily lives of individuals (by not delivering an intervention when the person is driving).

Although advances in mobile and wireless technology offer tremendous potential for delivering JITAIs, researchers often need more empirical evidence to inform the selection and adaptation of components in a JITAI, and these knowledge gaps can motivate scientific questions for randomized studies [e.g., (47–49)]. Consider *Example D* discussed above and suppose there is not yet sufficient evidence to determine (a) whether delivering a prompt that recommends a brief self-regulatory strategy is beneficial on average in preventing a lapse in the next 3 h, when individuals are not driving; and (b) what is the level of urge at which the prompt would be most beneficial. These questions concern the best component to deliver at different points in time in an intervention that intends to address conditions that change relatively rapidly. Hence, the answer to Question 5 would be “rapidly”, leading investigators to consider an MRT.

##### The Micro-Randomized Trial Design

Similar to the SMART, the MRT is a randomized trial that includes sequential randomizations (15, 16). However, MRTs include more frequent randomizations (relative to the SMART) because they are intended to provide data that investigators can use to answer questions about the selection and adaptation of components in a JITAI—an intervention that is motivated to address conditions that change rapidly. Accordingly, whereas the SMART is designed to answer questions about the longer-term effects of weeks or months of treatment on a distal outcome (e.g., an outcome measured at the month 6 follow up; Figure 3), the MRT is designed to answer questions about the short-term effects of relatively brief interventions on a proximal outcome (e.g., an outcome measured in the next 3 hours following a decision point).

**Figure 5:**
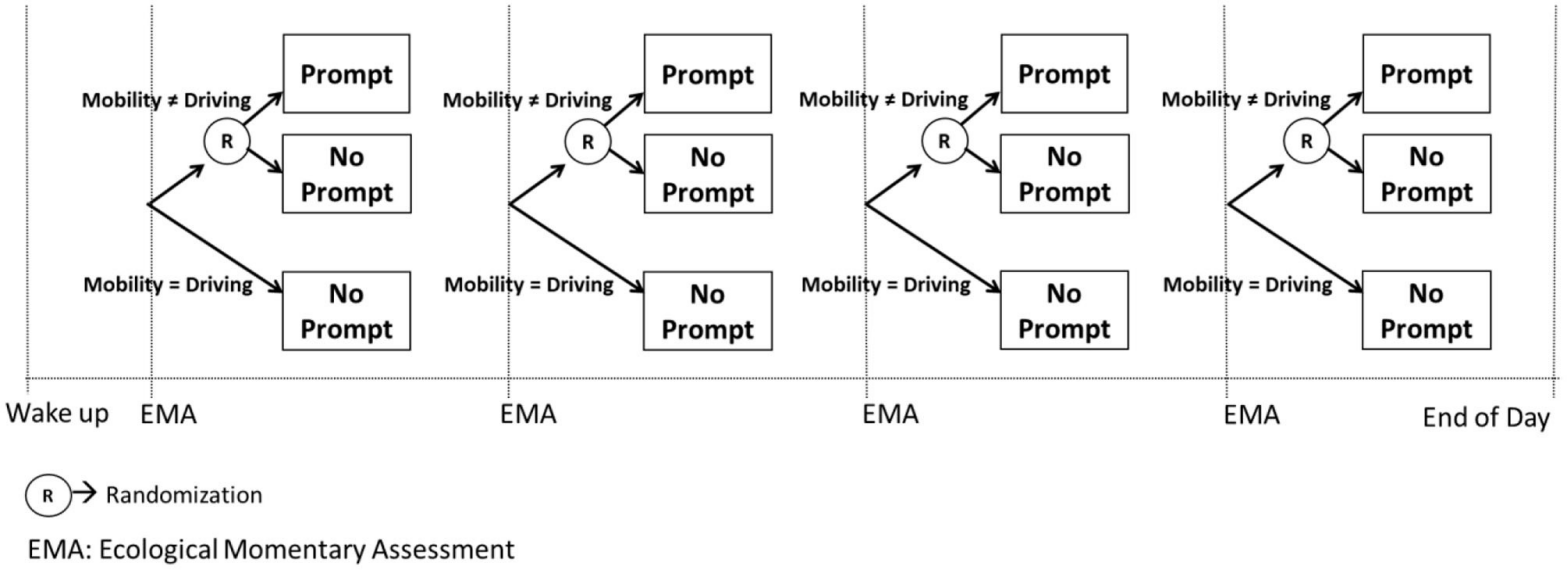
A MRT to answer Example E scientific questions.

For example, the MRT in Figure 5 is designed to answer the scientific questions outlined in *Example D*. Recall that these questions were: (1) whether delivering a prompt is beneficial on average in preventing a lapse in the next 3 hours when individuals are not driving; and (2) what is the level of urge in which the prompt would be most beneficial. To answer these questions, following each EMA which involves assessment of smoking urge, the person is randomized (with 0.5 probability) to either deliver a prompt or no prompt, provided that the person is not driving. If the person is driving a car, no prompt is delivered.

Similar to classic factorial designs and SMART designs, MRTs make extremely efficient use of study participants to answer questions about the selection and adaptation of components in a JITAI. This efficiency is facilitated by capitalizing on both between-subject and within-subject contrasts in the primary outcome (15, 50). For example, consider the first scientific question. Here, the primary outcome is whether or not the person experiences a lapse in the next 3 h. This (binary) outcome is assessed following each randomization. Hence, the first question can be answered by comparing two probabilities: (1) the probability of experiencing a lapse in the next 3 h when the individual was not driving and *a prompt was triggered* following the EMA, and (2) the probability of experiencing a lapse in the next 3 h when the individual was not driving and *a prompt was not triggered* following the EMA. This difference can be estimated by pooling data across all study participants and also across all decision points in which the individual was not driving (51). This is an estimate of the (causal) main effect of delivering a prompt (vs. no prompt), at decision points in which the individual is not driving.

The second scientific question can be answered by investigating whether the difference between the two probabilities described above varies depending on the levels of smoking urge (which was self-reported *via* the EMA immediately prior to randomization). That is, the data can be used to investigate whether the current level of urge moderates the causal effect of delivering (vs. not delivering) a prompt on the likelihood of lapse, provided that individuals are not driving. As before, this moderation analysis would use data across all study participants and across all decision points in which individuals were not driving (51). Estimates of the difference in lapse likelihood between delivering vs. not delivering a prompt at different levels of urge (e.g., low, moderate, and high) can be used to further identify the level(s) at which delivering (vs. not delivering) a prompt would be most beneficial.

Although the MRT is a relatively new experimental design, various types of MRT designs and analytic methods have been developed, allowing investigators to address scientific questions about the development of JITAIs. Examples include studies in which the proximal outcome is continuous (52) and binary (51), and studies in which the randomization probabilities are stratified to provide sufficient data to detect an interaction between the intervention options and a time-varying covariate (e.g., urge to smoke) (53). These methods enable investigators to leverage the MRT to inform the development of a wide variety of JITAIs.

## Discussion

This manuscript is intended to help investigators interested in developing a digital intervention select the most appropriate experimental design in light of their motivating scientific questions. Although existing tutorials can be used to guide the design and analysis of data from factorial designs [e.g., (11, 19, 20)], SMARTs [e.g., (22, 25)], and MRTs [e.g., (15, 16)], the selection of an appropriate experimental design remains an important challenge. The current framework emphasizes that specifying the scientific questions that are to be addressed is a critical prerequisite for selecting an experimental design. Although the examples provided in this manuscript focus on the development of a tobacco cessation intervention, the current framework is behavior agnostic. Thus, it can be used to inform not only tobacco cessation interventions, but also a wide variety of interventions.

### Limitations

There are several limitations to the current framework. First, for simplicity we focus on four types of experimental approaches. However, other types of experimental approaches [e.g., single-case designs (54), platform trials (55)] as well as non-randomized (observational) study designs (56) can be used to inform the development of digital interventions. Second, in much of this paper, we assumed that the scientific questions could be phrased in terms of a contrast between expected values of a single outcome between possible intervention options. However, a common challenge in intervention design is combining information from multiple goals and constraints, and often multiple stakeholders. A fruitful area for future methodological research would be analysis methods for more readily considering multiple outcomes or endpoints, and incorporating cost information (both monetary cost, or cost in time or effort) (57). Finally, the current framework does not provide guidance on how to address questions about the delivery of components *at different timescales*. Consider the following question: Under what conditions (e.g., level of urge, location) should a prompt that recommends a brief self-regulatory strategy be delivered to enhance therapeutic gains from weekly online quitting advice? This question concerns the synergy between components that are delivered at two timescales: (1) every few hours (prompts) and (2) weekly (quitting advice). Future research is needed to develop new experimental approaches and analytic methods that will enable scientists to investigate these types of potential synergies.

## Conclusion

While the current framework is not all-inclusive, it represents an important step in the development of clear guidelines for selecting study designs to inform the development of effective and scalable digital interventions.

## Data Availability Statement

The original contributions presented in the study are included in the article/supplementary material, further inquiries can be directed to the corresponding author.

## Author Contributions

IN-S served as a lead for conceptualization and writing. JD and DW contributed to reviewing, writing, and editing. All authors contributed to the article and approved the submitted version.

## Funding

This work was funded by awards from the National Institutes of Health: U01CA229437, P50 DA054039, R01 DA039901, and R01 CA224537.

## Conflict of Interest

The authors declare that the research was conducted in the absence of any commercial or financial relationships that could be construed as a potential conflict of interest.

## Publisher's Note

All claims expressed in this article are solely those of the authors and do not necessarily represent those of their affiliated organizations, or those of the publisher, the editors and the reviewers. Any product that may be evaluated in this article, or claim that may be made by its manufacturer, is not guaranteed or endorsed by the publisher.
